# Nurses’ knowledge to identify, prevent and manage hypertensive disorder of pregnancy

**DOI:** 10.4102/safp.v66i1.5995

**Published:** 2024-11-08

**Authors:** Princess Z. Mkhize, Vinogrin Dorsamy, Olive P. Khaliq, Jagidesa Moodley

**Affiliations:** 1 Department of Obstetrics and Gynecology, College of Health Sciences, University of KwaZulu-Natal, Durban, South Africa; 2 School of Laboratory Medicine and Medical Sciences, Faculty of Health Sciences, University of KwaZulu-Natal, Durban, South Africa; 3 Department of Pediatrics and Child Health, Faculty of Health Sciences, University of the Free State, Bloemfontein, South Africa; 4 Department of Obstetrics and Gynecology, Faculty of Medicine, University of KwaZulu-Natal, Durban, South Africa

**Keywords:** nurses, knowledge, practice, preeclampsia, eclampsia, low-and-middle-income-countries, hypertensive disorders of pregnancy, management

## Abstract

**Background:**

Hypertensive disorders of pregnancy are major contributors to maternal mortality in South Africa. Preventative strategies in low- and middle-income countries emphasise frequent antenatal visits, symptom identification, patient education and the prophylactic use of calcium and low-dose aspirin to prevent HDP for nurses because they are the frontline workers at antenatal clinics countrywide.

**Methods:**

This was a cross-sectional study where a self-administered questionnaire was conducted among nurses (midwives and professional nurses) employed at hospitals and clinics in Durban, South Africa, to assess their understanding and practices regarding identification and initial management of HDP, particularly for eclampsia and PE with severe features. The questionnaires were distributed in person by the researcher.

**Results:**

Of the 106 respondents, most (88.7%) worked in the public sector, with over 5 years of experience (64.9%). There was a varied understanding of HDP categories: 72.6% identified gestational hypertension correctly; 49.1%, chronic hypertension; 93.4% PE and 83.0% eclampsia. Knowledge of the recommended treatments for severe PE (55.7%) and eclampsia (66.0%) was moderate with respect to the recommended anticonvulsant and rapid-acting antihypertensive agents, with only 10% recognising the role of aspirin for the prevention of HDP.

**Conclusion:**

Substantial knowledge deficiencies existed among nurses in managing HDP.

**Contribution:**

Their crucial role in both emergency and preventative care in South African healthcare settings, enhancing educational training on clinical management by providing continuous training and regular updates are imperative to reduce maternal and perinatal complications associated with HDP.

## Introduction

Hypertensive disorders of pregnancy (HDP) contribute to maternal morbidity and mortality worldwide.^1^ Currently, 5%–10% of all pregnancies are affected by HDP globally.^2^ Hypertensive disorders of pregnancy are categorised into different classes, namely, chronic hypertension, white-coat hypertension, masked hypertension, gestational hypertension, preeclampsia (PE) and eclampsia.^3^ Preeclampsia and eclampsia are major health concerns globally, affecting maternal and perinatal wellbeing.^4^ These disorders are among the leading causes of maternal and perinatal morbidity and mortality, especially in low-to-middle-income countries (LMIC).^5,6^ In South Africa, HDP are the second leading direct cause of maternal deaths, accounting for 14.7% of maternal deaths between the years 2020 and 2022 according to the Saving Mothers Report, with most deaths attributed to the HDP categories PE with severe features and eclampsia.^7^ According to the sustainable development goal (SDG), countries are expected to abide by no more than 70 deaths per 100 000 live births by 2030.^7,8^

Preeclampsia is a multisystem condition characterised by high blood pressure (≥ 90 mmHg diastolic, ≥ 140 mmHg systolic) and proteinuria or evidence of end-organ system involvement, occurring after the 20th week of pregnancy. Preeclampsia also involves placental dysfunction, thus posing significant risks to both mother and foetus and requires careful clinical management and timely delivery. The exact causes are not fully understood, and PE is reported to occur in 5%–10% of pregnancies globally.^9^ However, it disproportionately affects women of African descent, primarily because of socio-economic factors rather than genetic predisposition.^10^ Risk factors of HDP include a previous history of PE, first pregnancy, unexplained stillbirths, multiple pregnancies, chronic hypertension, pregnancies arising from in vitro fertilisation, obesity and medical disorders such as diabetes, renal diseases and auto-immune conditions.^7^ Early clinical signs include excessive weight gain and oedema. The lack of advanced diagnostic tools in LMICs complicates early detection, emphasising the need for vigilant antenatal screening for clinical risk factors.^10^ This may result in severe clinical features such as a blood pressure (BP) reading of systolic blood pressure of ≥ 160 mmHg and a diastolic ≥ 110 mmHg with proteinuria dipstick measurement of +2 and above (ISSHO 2021). Therefore, clinical history and examinations are used to establish whether women are more likely to develop HDP. Furthermore, most women initiate antenatal care at primary healthcare centres (clinics and district hospitals) and community healthcare centres in South Africa. Nurses play a vital role in the South African healthcare system, especially at the primary care level. Nurses are typically the first point of contact for pregnant women, positioning them as key players in the early identification and management of HDP. Their responsibilities extend to recognising women at risk and managing severe cases of PE or eclampsia until appropriate referral to specialised care can be made.

Given the dynamic nature of clinical guidelines and frequent staffing changes at health clinics, there is a vital need to regularly assess and update the training of nurses. Their readiness and competence in managing HDP are crucial for enhancing patient outcomes and reducing maternal and infant mortality rates associated with these conditions.

This study seeks to evaluate the knowledge and skills of nurses in the identification and initial management of HDP in South Africa. The skills were evaluated based on the knowledge of appropriate measurement of BP, using the correct cuff size for different body mass index (BMI), accurate measurements of weight, protenuria and ensuring that there is no oedema.

The objectives of this study were as follows:

- To assess the current level of knowledge among nurses and midwives regarding the clinical characteristics and management protocols for HDP.
- To identify any gaps in knowledge that may affect the quality of care provided to patients with HDP.
- To provide recommendations for targeted educational and training programmes that could help bridge these knowledge gaps and enhance the efficacy of HDP management.

## Research methods and design

### Study design and participants

A prospective, cross-sectional study was conducted utilising a self-administered, structured questionnaire specifically developed for this research. To test for the validation of the questionnaire, a pilot study with a maximum of 10 questionnaires was conducted. Nurses, specifically midwives and professional nurses (nurses with a qualification) working at regional hospitals, district hospitals, private hospitals and primary health clinics in a single geographical area, Durban, South Africa, were invited to participate. The sample size was 200 participants with a statistical power of 80%, using the Cohens effect. However, only 106 nurses returned the questionnaires. The researcher approached the hospital and/or clinic manager and presented the study. Permission to interview the nurses was granted verbally by the manager and the researcher distributed hard copies of the questionnaire to all nurses willing to participate, and the questionnaires were collected after 2 days. The study focussed on assessing their knowledge and practices related to the initial management of HDP, with a particular emphasis on PE and eclampsia.

### Questionnaire development and distribution

The questionnaire was designed by the research team to evaluate both theoretical knowledge and practical applications in managing HDP.

### Data collection

The questionnaire included sections on defining hypertensive disorders, recognising symptoms and risk factors, and identifying initial treatment steps for conditions like severe PE and eclampsia. Some questions were multiple choice structured questions while some included ‘yes/no’ questions. The questionnaire consisted of 47 questions. The questionnaire was distributed in person to all midwives and professional nurses who were willing to participate in the study. The midwives and professional nurses were given a period of 2 days to fill out the questionnaires to allow them to answer the questions comfortably and to allow them to fulfil their daily duties. To protect the identity of the participants, the questionnaires were filled out anonymously. The data collection was commenced from July 2021 to July 2022. Responses were collected in person by the researcher after 2 days and securely stored in a lockable cupboard. The data were captured on a password-protected spreadsheet for analysis. The operational definition of HDP employed for this study included gestational hypertension, PE, severe features of PE and eclampsia as categorised by specific clinical criteria as follows:

- Gestational hypertension; hypertension (BP ≥ 140/≥ 90 mmHg) appearing for the first time after the 20th week of gestation.
- Chronic hypertension is defined as women with a history of hypertension.
- Preeclampsia is defined as hypertension (BP ≥ 140/≥ 90 mmHg) appearing for the first time after 20 weeks of pregnancy and associated with laboratory signs of organ affectation.
- Preeclampsia with severe features such as a BP of > 160/110 mm Hg and/or severe headaches, nausea and vomiting, blurring of vision and epigastric pain.
- Eclampsia, seizures associated with signs of PE.

### Statistical analysis

Collected data were analysed quantitatively using descriptive statistics to quantify the knowledge levels and practice patterns among the participants. The analysis aimed to highlight areas of strength and identify critical gaps where additional training or resources are needed. Data were analysed using GraphPad Prism, V 5.03 (Graph-Pad Software Inc, CA, United States).

### Ethical considerations

Ethical approval, from the Biomedical Research Ethics Committee at the University of KwaZulu-Natal (BREC: 136/19) and provincial approval from the Department of Health were obtained. After obtaining informed consent, the questionnaires were distributed to all nurses who agreed to participate, ensuring minimal interference with their daily responsibilities. Participants completed the questionnaires anonymously and independently and returned them to the primary investigator upon completion.

## Results

### Socio-demographic characteristics of respondents

A total of 200 questionnaires were distributed to all nurses who were willing to participate in the study. Only 106 questionnaires were returned. The response rate was 53%. The ones that did not return the questionnaires were lost to follow-up (*n* = 94). The total sample size used in the study was 106. The questionnaire included 106 nurses predominantly employed in the public health sector (88.7%), with a smaller representation from the private sector (11.3%). To overcome bias, two district office nurses were also recruited to validate the responses given by the nurses. However, some of the nurses (*n* = 4) did not respond to questions pertaining to procedures of blood pressure measurement and the assessment of oedema; therefore, they were not included in the applicable questions. The gender distribution was predominantly female, that is, 90 out of 106 (84.9%). The mean (s.d.) age of the respondents was 41.4 ± 8.6 years. Most nurses (44.3%) had 6–10 years of experience (Table 1).

**TABLE 1:** Socio-demographic and clinical characteristics of all participants.

Variable	Number of observations (*N* = 106)	Frequency (%)
**Response rate (*N* = 200)**	106	53
**Employment facility**		
Clinic	46	43.4
District hospital	3	2.8
Regional hospital	43	40.6
Private hospital	12	11.3
District office	2	1.8
**Age (years) groups**		
< 30	17	16.0
31–40	32	30.2
41–50	41	38.7
51–60	12	11.3
> 60	1	0.9
**Sex**		
Male	13	12.3
Female	90	84.9
**Nurses: years of experience**		
< 2 years	8	7.5
3–5 years	12	11.3
6–10 years	74	44.3
> 10 years	42	39.6

Note: Age (years): mean ± s.d. = 41.3 ± 8.6.

Table 1 categorises participants by sector of employment, experience level and biological sex, highlighting the diversity and background of the surveyed group.

### Hypertensive disorders of pregnancy

Knowledge assessments revealed varied understanding across different categories of HDP. Out of 106 nurses, 72.6% defined gestational hypertension correctly. Notably, PE/eclampsia (93.4%, 83.05%) was better understood compared to chronic hypertension (49.1%) (Table 2).

**TABLE 2:** Knowledge of hypertensive disorders by category (*N* = 106).

Hypertensive disorder of pregnancy	Correctly identified	%
Gestational hypertension	77	72.60*
Chronic hypertension	52	49.10
Preeclampsia	99	93.40
Eclampsia	88	83.05

Table 2 displays the percentage of respondents who correctly identified one or more types of hypertensive disorder, illustrating areas of strength and potential gaps in knowledge.

### Procedure of blood pressure measurements in pregnancy

Skills were accessed in the following terms. In terms of the appropriate patient position for reading BP, 102 out of 106 (96.2%) participants preferred to measure BP with the woman sitting on a chair. Regarding the different sizes of arm cuffs to measure BP, 63 out of 106 (59.4%) nurses indicated that there were small, medium and large cuffs. Approximately 23 out of 106 nurses (21.7%) indicated small and large cuff, 3 out of 106 nurses indicated (2.8%) medium and large cuff, and 1 out of 106 nurses (0.9%) indicated a large cuff.

### Assessment of oedema

One hundred and two (96.2%) of the participants responded that they would assess oedema in pregnant women as a sign of HDP. Seventy eight point four per cent of the 102 participants defined oedema assessment in terms of grading from 1-3 (1 = mild, 2 = moderate, 3 = severe). Twelve point seven per cent defined oedema assessment in grades 1-4 (1 = mild, 2 =moderate, 3 moderate to severe, 4 = severe) while 14.7% defined the assessment in terms of mild, moderate to severe, without the use of grades.

### Preeclampsia

Ninety-nine (93.4%) defined PE correctly.

### Use of hypertensive disorders of pregnancy guidelines

All the midwives and professional nurses (*n* = 106) had a positive view of the use of clinical guidelines on HDPs. The nurses were asked if they use guidelines given to them to define HDP. Only 101 participants answered ‘yes’ to the question. Of the 101, 97 nurses used copies available at the facility (96.0%), while 73 (72.3%) knew that the guidelines were updated regularly. Of the total number of participants (*n* = 106), 40 (24.4%) had access to HDP guidelines through written forms at the clinic, and some had access through posters (23.3%), during training (7.3%) and computers (21.3%). In this question, one nurse had access to HDP guidelines in more than one way. Therefore, in Table 3, the answers provided for each question may exceed the total number of participants.

**TABLE 3:** Different aspects of hypertensive disorder of pregnancy guidelines: Nurses’ knowledge of the existing guidelines on the management of hypertensive disorders of pregnancy (*N* = 106).

Different aspects of HDP guidelines	Number of observations	%
**Use of guidelines to define HDP**	101	95.3
Copies of guidelines available at a facility	97	96.0
Guidelines updated regularly	73	72.3
**Access to HDP guidelines**		
Written form at clinic	40	24.4†
Poster	38	23.2
Appt	12	7.3
Computer	35	21.3
HDP guidelines are discussed regularly at perinatal/review meetings.	39	23.8
**Action taken if no written HDP guidelines available**		
Scheduled visit within 3–5 days	13	11.3†
Refer to doctor.	45	39.1
Refer to the next level of care.	33	28.7
Severity of condition	24	20.9
**Referral pattern of PE patients**		
Referred patients with any type of HDPs	59	55.6
Level of healthcare referred to:		
District hospital	10	9.4†
Regional hospital	45	42.5
Tertiary hospital	4	3.8
Referral indicators present at the facility	49	46.2
Training on referral patterns and indicators available at the facility	37	34.9

HDP, hypertensive disorders of pregnancy; PE, preeclampsia.

† , The percentages do not add up to 100% as there were one or more responses to different aspects of nurses’ knowledge of the existing questions on the management of HDPs.

Nurses who work at the clinic were asked about their referral pattern of patients with PE. Only 59 (55.6%) of the nurses claimed that they do referrals. Most nurses claimed that they refer patients to regional hospitals (*n* = 45), and only four nurses referred to tertiary hospitals. A total of 49 nurses knew of referral indicators present at the facility, while 37 nurses knew of the availability of training on referral patterns and indicators at the facility (Table 3).

Table 4 is on the knowledge of clinical manifestations of PE with severe features. In terms of blood pressure, a total of 58 (54.7%) of the nurses knew of the different measures of blood pressure that indicate severity. The number of nurses who knew the different BP measures are stated in Table 4. Of the 106 nurses, 66 (62.2%) knew that protein in the urine was an indication of severe disease. In terms of signs and symptoms, 36.8% knew of epigastric pain, blurred vision and headache, and 14.2% knew of epigastric pain, blurred vision, headache and oedema. Only 30 nurses responded to the question on blurred vision and headache with 3 (2.8%) who knew of both blurred vision and headache, 3 (2.8%) who knew of blurred vision alone, and lastly 3 (2.8%) who knew of a headache only.

**TABLE 4:** Knowledge of clinical manifestations of pre-eclampsia with severe features.

Parameters	Total number of participants	Numbers of observations	%
**Blood pressure (mmHg)**			
160/110	106	28	26.4
160/100	106	26	24.5
≥ 140/90	106	4	3.8
**Proteinuria**			
= 1	106	39	36.8
≥ 2	106	27	25.5
**Signs and symptoms**			
Epigastric pain, blurred vision and headache	106	39	36.8
Epigastric pain, blurred vision, headache and oedema	106	15	14.2
Blurred vision and headache	106	16	15.1
Blurred vision	10	3	2.8
Headache	10	3	2.8

### Laboratory investigations for pre-eclampsia

Ninety-nine (93.4%) respondents indicated that laboratory tests are necessary for PE. The blood and urine tests are listed in Table 5.

**TABLE 5:** Response rates on the necessity of laboratory tests during pregnancy.

Investigation	Number of observations	%
FBC and U&E	1/99	1.01
FBC and U&E & LFT	26/99	26.3
FBC and U&E & LFT and Urine	40/99	40.4
FBC and U&E & LFT and 24-h Urine	32/99	32.3

FBC, full blood count; U&E, urea and electrolytes; LFT, liver function tests.

Data in Table 6 shows the distribution of the nurses (*n* = 106) who had knowledge of the best anticonvulsant for eclampsia and the rapid-acting antihypertensives; 55.7% and 66.0% of all midwives gave the correct answer for the best anticonvulsant and choice of rapid-acting antihypertensive, respectively.

**TABLE 6:** Choice of anticonvulsants/rapid-acting antihypertensive and side effects of magnesium sulphate (*N* = 106) listed by participants.

Variables	Number of observations	%
**Choice of anticonvulsants**		
MgSO_4_	59	55.7
MgSO_4_ and diazepam	11	10.4
MgSO_4_ and l (clonazepam)	11	10.4
Diazepam	7	6.6
(clonazepam) alone	4	3.8
**Choice of rapid acting antihypertensive agents**		
Nifedipine	70	66.0
Nifedipine and labetalol	12	11.3
Nifedipine, labetalol and hydralazine	1	0.9
Nifedipine and hydralazine	2	1.9
Aldomet	8	7.5
Labetalol	1	0.9
**Side effects of MgSO_4_**		
Dyspnoea	21	19.8
Dyspnoea and low BP	18	17.0
Low BP	13	12.3
No reflexes	11	10.4
Dehydration and confusion	10	9.4

MgSO_4_, magnesium sulphate.

### Eclampsia box (A prepared box containing all the required drugs and equipment for the emergency management of preeclampsia with severe features and eclampsia)

Seventy-one (66.9%) midwives were aware of an eclampsia box. Thirty-nine (36.8%) knew about the contents of an eclamptic box.

### Aspirin use in pregnancy

Thirty-three (31.1%) midwives knew aspirin use in pregnancy (Table 7).

**TABLE 7:** Knowledge of the use of aspirin to prevent hypertensive disorders in pregnancy during preeclampsia.

Indication	Number of observations	%
Prevent preeclampsia	12	11.30
Reduces bleeding	14	13.20
Prevents foetal abnormalities	6	5.70
Decreases high blood pressure levels	1	0.94

## Discussion

This study’s findings underline considerable knowledge gaps among nurses managing HDP, particularly in the emergency management of pre-eclampsia and eclampsia. While most participants demonstrated a foundational understanding of these conditions, critical deficiencies in knowledge about preventative measures and emergency management protocols were apparent. These findings are consistent with those reported by the study conducted in LMICs, which found that healthcare providers often lack comprehensive training on HDP, impacting the quality of care delivered.^11^ Despite their extensive experience (> 10 years), significant gaps in knowledge were observed, similar to findings in the studies from other countries. For instance, only 49.1% could correctly define chronic hypertension in the current study, a key risk factor for pre-eclampsia, comparable to findings by the study in the Western Cape that reported that 70.3% of nurses defined chronic hypertension correctly. In contrast, lower rates in a Romanian study were reported (9.2%).^12,13^ Another study in Tanzania found that 73.3% of the nurses had excellent knowledge of the management of PE.^14^ An earlier study revealed that in terms of the normal range of blood pressure, 98% were highly knowledgeable and 92% were found knowledgeable in the causes of hypertension during pregnancy.^15^ Regarding pre-eclampsia and eclampsia, a study found that midwives and doctors caring for pregnant women with pregnancy-induced hypertension have limited knowledge about pre-eclampsia/eclampsia.^14^ A recent study from Saudi Arabia reported that the public health nurses were highly knowledgeable (3.29%) in the definition of hypertension in pregnancy as well as inappropriate patient positioning for current readings of blood pressure (weighted mean of 3.37). These inconsistencies highlight ongoing challenges in standardising knowledge across different settings.

The clinical guidelines from the South African National Department of Health were reportedly used among surveyed midwives, with 95% utilising these resources in various formats such as written guidelines, posters and digital files. Despite high guideline usage, only 68.9% reported that these guidelines were regularly updated, and 56.6% discussed them at perinatal meetings, suggesting potential lapses in guideline dissemination and engagement. The knowledge of clinical guidelines on the treatment of PE by midwives is essential so that women with an HDP especially PE with severe features or eclampsia can be provided immediate management and refer them early to the regional or tertiary levels of healthcare for further management. However, a study at the Eastern Cape Province clinics in South Africa found that midwives lack the knowledge to manage PE.^13^

A strong emphasis in the current study was placed on the correct technique for measuring blood pressure, deemed essential for diagnosing hypertension during pregnancy because there were reports of the unavailability of different BP cuff sizes and the technique used to measure BP in pregnancy. Most respondents preferred to measure blood pressure with the patient seated, using appropriately sized cuffs. However, there was variability in the usage of different cuff sizes, which could impact the accuracy of measurements.

Laboratory investigations for severe PE indicated that while more than half of the respondents recognised symptoms like epigastric pain and blurred vision, a significant proportion failed to identify all relevant symptoms, which could hinder effective management. This finding aligns with a previous study, which also reported shortcomings in identifying clinical manifestations of PE with severe features.^15^

Regarding treatment, 92 (86.7%) of the nurses knew the correct anticonvulsants recommended for use to prevent or treat seizures in women experiencing PE with severe features or eclampsia. These findings were similar to those that showed that 95% of nurses knew of the use of magnesium sulphate, methyldopa and hydralazine to treat PE.^16^ In the current study, nifedipine and labetalol were frequently used as rapid-acting anti-hypertensive, and magnesium sulphate was commonly employed as an anticonvulsant for severe PE, reflecting a general adherence to recommended pharmacological treatments. However, the role of aspirin in preventing PE remains underappreciated, with only a small percentage (31.1%) of nurses aware of its preventive potential, despite evidence supporting its efficacy in reducing PE incidence.^17^ However, our results are slightly higher compared to the results of a study conducted in the Eastern Cape, South Africa which found that only 28.9% of nurses knew that aspirin can be used as a preventative measure of HDP.^15^ On the other hand, a Malawian study indicated that only one of seven nurses knew that low-dose-aspirin may be used to prevent PE.^18^ It is quite possible that the use of low dose aspirin is not widely known to reduce the frequency of HDP because the guidelines in South Africa do not firmly state its usage and that most women have their first antenatal visit after the 14th week of pregnancy. It is generally recommended that aspirin be initiated prior to 14 weeks gestational age.^10^

The knowledge of referral indicators and training on referral patterns was only reported by a minority, illustrating a need for improved training in referral practices to ensure timely and effective patient management, especially in clinical settings, where medical doctor presence is rare. The results of the current study differ from a study published in the Eastern Cape, South Africa that showed that 52.5% of the nurses were aware of the indications and timing of referral in women with HDP.^19^

Additionally, the moderate familiarity with tools such as the ‘eclampsia box’ suggests a potential shortfall in readiness for acute management of PE with severe features and eclampsia, which is concerning given the high numbers of such emergencies. This finding aligns with the conclusions of Magee et al., who emphasised the importance of practical training in emergency obstetric care to improve outcomes in settings burdened by HDP.^18^

In summary, while there is substantial adherence to clinical guidelines and a high level of experience among nurses in KwaZulu-Natal Province, critical gaps in knowledge and practice remain, particularly in the areas of definition of HDP, symptom recognition and preventive medication usage. Addressing these gaps through updated training programmes and regular guideline reviews is essential to enhancing the management of hypertensive disorders in pregnancy.

### Strengths and limitations of the study

The study revealed important discrepancies between the knowledge levels of nurses and the best practices outlined in national, local and international guidelines for the management of HDP. Our study has several limitations that should be considered when interpreting the results. The reliance on a convenience sample from a single geographical area may limit the generalisability of the findings to other regions or healthcare settings within South Africa. Additionally, the study’s design, based on self-reported measures, could lead to response bias where participants might either overestimate or underestimate their actual knowledge and practices because of social desirability or recall biases. Furthermore, nurses did not answer the questions they did not know the answers to which accounted for missing information which accounted for the varying numbers in the tables.

### Implications for future directions

Future studies could benefit from a mixed-methods approach that includes direct observations or simulations to assess the practical skills of healthcare providers. Incorporating qualitative components could also provide deeper insights into the reasons behind the knowledge gaps and barriers to guideline adherence.

## Conclusion

Based on this research’s findings, knowledge and practice levels were inadequate. The nurses should not only be updated in theoretical knowledge but their clinical skills should also be updated to ensure competence. Nurses must be qualified in the advanced optimal care of obstetric emergencies, including continuous refresher courses to ensure competence in theory and practice for all practising midwives.
